# Coating Cutting Blades with Thin-Film Metallic Glass to Enhance Sharpness

**DOI:** 10.1038/s41598-019-52054-3

**Published:** 2019-10-29

**Authors:** Jinn P. Chu, Wahyu Diyatmika, Yong-Jhe Tseng, Yu-Kang Liu, Wen-Che Liao, Shih-Hsin Chang, Ming-Jen Chen, Jyh-Wei Lee, Jason S. C. Jang

**Affiliations:** 1 Department of Materials Science and Engineering, National Taiwan University of Science and Technology, Taipei, 10607 Taiwan; 2 Applied Research Center for Thin-Film Metallic Glass, National Taiwan University of Science and Technology, Taipei, 10607 Taiwan; 3 Department of Surgery, Mackay Memorial Hospital, Taipei, 10449 Taiwan; 4 Department of Medicine, Mackay Medical College, New Taipei City, 25245 Taiwan; 5 Department of Materials Engineering, Ming Chi University of Technology, New Taipei, 24301 Taiwan; 6 Department of Mechanical Engineering, National Central University, Taoyuan, 32001 Taiwan

**Keywords:** Preclinical research, Surfaces, interfaces and thin films

## Abstract

In this study, we sought to enhance the cutting properties of the various blades by coating them with Zr- and Fe-based thin film metallic glasses (TFMGs) to a thickness of 234–255 nm via sputter deposition. In oil-repellency/sliding tests on kitchen blades, the sliding angle and friction forces were as follows: bare blades (31.6°) and (35 µN), Ti-coated blades (20.3°) and (23.7 µN), and Z-TFMG coated blades (16.2°) and (19.2 µN). Comparisons were conducted with bare blades and those with a Teflon coating (a low-friction material commonly used for the coating of microtome blades). We also found that the Teflon coating reduced the cutting forces of an uncoated microtome blade by ~80%, whereas the proposed Z-TFMG achieved a ~51% reduction. The Z-TFMG presented no indications of delamination after being used 30 times for cutting; however, the Teflon coating proved highly susceptible to peeling and the bare blade was affected by surface staining. These results demonstrate the efficacy of the TFMG coating in terms of low friction, non-stick performance, and substrate adhesion. The performance of Z-TFMG and F-TFMG was also evaluated in split-thickness skin graft surgery using dermatome blades aimed at elucidating the influence of TFMG coatings on the healing of surgical incisions. When tested repeatedly on hairless skin, the surface roughness of uncoated blades increased by approximately 70%, whereas the surface roughness of TFMG-coated blades increases by only 8.6%. In the presence of hair, the surface roughness of uncoated blades increased by approximately ~108%, whereas the surface roughness of TFMG-coated blades increases by only ~23%. By Day 7, the wounds produced using TFMG-coated blades were noticeably smaller than those produced using uncoated blades, and these effects were particularly evident in hairy samples. This is a clear demonstration of the efficacy of TFMG surface coatings in preserving the cutting quality of surgical instruments.

## Introduction

Skin grafts involve the removal of skin from one area of the body for transplantation in another area of the body. This technique is commonly employed when skin is damaged due to extensive trauma, such as burns. The procedure dates back to 1869 when J. L. Reverdin succeeded in applying epidermal grafts to a granulating wound^1^. This approach to wound coverage has greatly advanced since that time, and is now considered a safe, reliable, and practical procedure. Skin grafts can be categorized as split-thickness and full-thickness. Split-thickness grafts are used to cover large areas, and feature a low rejection rate^2^. One key aspect of reconstructive and plastic surgery relates the process of preparing the corresponding donor sites and the actual process of splitting the skin. Blades of higher sharpness, strength, and durability tend to be more accurate in the removal of damaged skin or the harvesting of new grafts. It is for this reason that most surgical blades are composed primarily of martensitic stainless steel (AISI 420), which has high hardness as well as good resistance to wear and mechanical deformation at the tip of blade. Surgical scalpels and blades of martensitic stainless steel are standard tools in soft tissue surgery^3^. Wear resistance is particularly important when making long incisions, often reaching 30 cm or longer. The polycrystalline structure of martensitic stainless steel includes grains and grain boundaries, which unfortunately serve as crack initiation sites, eventually leading to localized fracturing on the blade. These effects can be seen in the irregular, wavy surfaces at edge-tip of surgical blades under magnification^4^. The application of a dermatome to cut soft tissue of skin can produce tearing trauma, which slows wound healing and increases the pain associated with surgery^5^. Furthermore, it is not economically feasible to use disposable stainless steel scalpels, which lose their sharpness rapidly and thus necessitate the use of multiple devices to perform a single surgical incision. Numerous coatings have been proposed to overcome this issue. Pini *et al*.^6^ reported on a variety of hard coatings for stainless steel surgical scalpels, including diamond-like carbon (DLC), tungsten carbide/carbon (WC/C) and titanium nitride (TiN). Their results indicate that these coatings are well suited to reusable surgical scalpels.

Nonetheless, most surgeons encounter considerable resistance when attempting to cut hair tissue (hair follicle and root sheath), which can result in damage to the blade. The donor site can be hairless or highly hairy depending on its location, which is also affected by ethnic variation. Body hair is generally removed from donor sites prior to the skin grafting procedure; however, complete removal is not always possible. Furthermore, a failure to remove hairs can increase the risk of infection and/or result in damage to the blade^7,8^.

Sharp blades were shown to create incisions of higher quality than those obtained using blunt blades, thereby requiring less cutting force and inducing less pain for the patient^3^. In a previous study, we modified the surface of the blade by applying thin film metallic glass (TFMG) coating to provide a smooth surface, high hardness, and antibacterial properties. This was done primarily to enhance the sharpness of the surgical instruments and prevent the transmission of bacteria^9–12^. TFMG with a low coefficient of friction (COF) of ~0.05 is a promising material to replace silicone fluid lubricants on the surface of medical needles for reducing the insertion force^12^. By contrast, the hardness and COF of TFMG coating are superior to those of martensitic stainless steels, exhibiting hardness of ~5.5 GPa and COF of ~0.55–0.80^13–15^.

These characteristics have made TFMG an ideal candidate for the coating of stainless steel surgical blades^16–18^. It has been reported that Zr-based TFMG coating improves the sharpness of dermatome blades, thereby reducing surface roughness and producing incisions of greater smoothness^9,19^. Jang *et al*.^20^ reported an Fe-based TFMG coated blade with cutting durability superior to that of a commercial blade, which maintained a sharp edge-tip after cutting over a distance of 30 cm. Recently, we also reported the insertion of TFMG-coated needles was shown to reduce the trauma of the porcine tissue by ~44%^21^. Here, we extended these works by employing TFMG-coated dermatome blades to split-thickness skin graft surgery involving the skin of a live pig. We also investigated the performance Fe-based TFMGs^22–24^ on dermatome blades. Our aim was to promote the healing of wounds induced through surgery and investigate the influence of hair in these procedures. We measured the degree to which the TFMG coatings reduced the friction force and cutting force, as an indicator of the degree to which the coating enhanced the sharpness of the blades. The proposed TFMG coatings were deposited on kitchen knives and microtome blades to assess cutting performance and oil-repellency. Cooking knives were selected because they are commonly used to cut greasy substances and non-stick surfaces are preferred to facilitate cleaning and prevent cross-contamination among foods. Microtome blades were chosen because they are used to cut biological samples into extremely thin slices to facilitate observation via light transmission or electron radiation. Microtome blades are commonly coated with a layer of Teflon to reduce surface friction during cutting. In this study, a TFMG coating was applied to bare microtome blades (i.e., without a Teflon coating). Dermatome blades were prepared to assess the effectiveness of TFMG coatings in skin grafting procedures.

## Materials and Methods

### Coating depositions and characterizations

**Figure 1:**
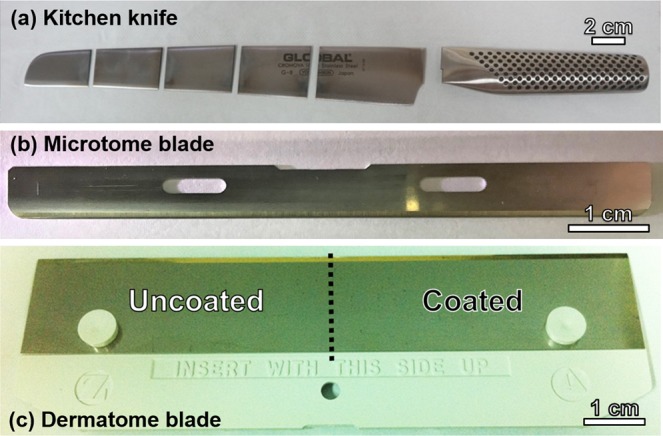
(**a**) Kitchen knife, (**b**) microtome, and (**c**) dermatome blades used as the substrates in this study. Half of the dermatome blade surface was coated with TFMG and the other half remained uncoated.

High-quality kitchen knives (Yoshikin G18) and S35 microtome blades (manufactured by Feather) were used as substrates to assess the performance of the TFMG coatings in terms of oil-repellency and cutting performance, as shown in Fig. 1(a,b), respectively. A dermatome blade (manufactured by Zimmer) was used for the skin grafting procedure, as shown in Fig. 1(c). Note that half of the dermatome blade surface was coated with TFMG and the other half remained uncoated. The dotted line in Fig. 1(c) indicates the division between the two areas.

Zr_53_Cu_33_Al_9_Ta_5_ and Fe_65_Ti_13_Co_8_Ni_7_B_6_Nb_1_TFMGs in atomic percent, (hereafter denoted as Z-TFMG and F-TFMG, respectively) were deposited on the blades via radio frequency (RF) magnetron sputtering, using RF power of 100 W, substrate bias of 100 V, base pressure of <2 × 10^−6^ Torr, and working pressure of 3 mTorr. The Z- and F-TFMG coatings have thicknesses of 255.4 ± 6.2 nm and 234.0 ± 0.5 nm, respectively. Pure Ti and Teflon were also coated on the blades for comparison. The hardness of the TFMGs was characterized via nanoindentation using a TI 950 TriboIndenter (Bruker). The characteristics of Z-TFMG and F-TFMG of the same composition have been detailed in numerous studies^9,25^.

### Stickiness characterizations

#### Oil-repellency test

The oil-repellency of the Z-TFMG and pure Ti coatings was evaluated by studying the interaction between fish oil and the underlying substrate of kitchen knives. The as-received blade was sectioned via wire-cutting into five pieces (length of 40 mm per piece) to ensure uniformity in the film after sputter deposition (see Fig. 1(a)). Test samples were placed on the stage of a contact angle meter (Sindatek Model 100SB), whereupon fish oil (MADRE LABS; contents: anchovy, mackerel, sardine; density: 0.97 mg/µl) was applied drop-wise on the surface of the samples. As the stage was gradually tilted, a camera was used to monitor the shape of the droplet and record the angle associated with each frame. The angle associated with the first image presenting evidence of sliding was defined as the sliding angle. The sliding angle was then used to estimate the friction forces, under the assumption that the rotation momentum and deformation of the droplets were disregarded. The equation used to calculate the friction forces was as follows:

1 $${\rm{Friction}}\,{\rm{force}}={\rm{volume}}\,{\rm{of}}\,{\rm{droplet}}\times {\rm{density}}\,{\rm{of}}\,{\rm{fish}}\,{\rm{oil}}\times \,\sin \,{\rm{\theta }}\times 9.8$$

where θ is the sliding angle, and the density of the fish oil was 0.97 mg/µl. A micropipette was used to ensure that the volume of each fish oil droplet on the blade was 7 µL. All measurements were repeated at least 10 times for each sample. The samples were thoroughly cleaned between tests.

#### Cutting tests

The effect of the TFMG coating on cutting performance was evaluated through the insertion and retraction of the microtome blade with TFMG coating into pig muscle using a material test system (MTS 370). The insertion and retraction forces were measured at a speed of 0.5 mm/sec over a cutting distance of 20 mm. Cubes of pig muscle with the average thickness of 30 mm were obtained from the hindquarters of pig flesh, which was purchased from a supermarket and inspected by the Certified Agricultural Standards (CAS) in Taiwan. At least five bare blades, five Teflon-coated blades, and ten Z-TFMG coated blades were evaluated to confirm the validity of the data.

### Animal test

Animal testing was conducted after receiving approval by the faculty of Mackay Memorial Hospital Commission for Animal Testation (approval number: MMH-A-S-103–12). All animal test experiments were executed in accordance with relevant guidelines and regulations. Split-thickness skin graft surgery was performed on the backs of 6-month-old female pigs under general anesthesia. Anesthesia was induced using Atropine and Ketamine, followed by the intravenous injection of Thiopental (1 mg/kg). The entire back of each pig was shaved and prepared using antiseptic solution (10% povidone iodine), and then sterilely draped with the pig in prone position. Split thickness skin grafts (15/1000 inch in thickness) were harvested over a distance of 50 cm (hairless skin) and 10 cm (hairy skin). Before and after animal experiments, we examined the morphology of the coated and uncoated blades along the edges and on the surfaces using a scanning electron microscope (SEM, FEI Quanta 3D FEG) operated at a working distance of 10 mm under accelerating voltage of 10 kV. The surface roughness of the samples was measured using a non-contact 3D surface profiler (coherence correlation interferometer, CCI, Taylor Hobson) with green LED source. Average surface roughness (root mean square, RMS) was calculated from five areas near the edge of the blade (100 µm × 400 µm each). When the skin grafting procedure was completed, the wounds were coated with neomycin and bacitrazin ointments, then dressed with gauze bandages. The pig was then returned to its cage, where it was monitored until spontaneous and purposeful movement was observed. During this initial observation period, acetaminophen powder was administered in its drinking water for the relief of pain.

To monitor wound healing, we performed an additional skin grafting procedure on the back of the same pigs covering treatment areas of 4 cm (length) by 2 cm (width). After the harvesting of skin, the wound was covered with artificial skin (Duoderm^®^) to prevent infection. A digital camera was used to continuously record the process of wound healing from the first day (Day 1) to the seventh day (Day 7). To elucidate the effects of cutting distance in skin graft surgery, we created a 30-cm long incision on the back of the pig under hairless conditions. A digital camera was then used to record wound healing over a period of 7 days.

## Results and Discussion

### Oil-repellency and cutting performance of TFMG coating

**Figure 2:**
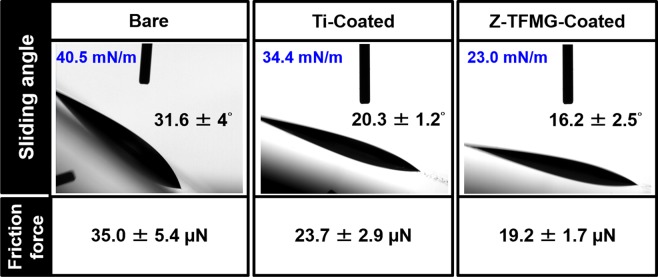
Sliding angle and friction force measurement results of bare, Ti-, and Z-TFMG-coated blades. Surface free energy (SFE) in blue font for different surface conditions are obtained from^12^.

Figure 2 presents the results of the oil-repellency/sliding tests on kitchen blades in terms of sliding angle and friction forces: bare blades (31.6°) and (35 µN), Ti-coated blades (20.3°) and (23.7 µN), and Z-TFMG coated blades (16.2°) and (19.2 µN). The oil repellency of the Z-TFMG coating clearly exceeded that of the other surfaces, which indicates a ~45% reduction in the friction forces associated with oil sliding on the bare blade. Figure 2 also lists the surface free energy (SFE) of the three samples, as follows: bare blade (40.5 mN/m), Ti-coated blade (34.4 mN/m), and Z-TFMG coated blade (23.0 mN/m). The details of these SFE measurements have been published elsewhere^12^ and for the sake of brevity are not discussed here. This trend is consistent with the sliding angle and the friction force measurements, indicating that the decrease in sliding angle and friction force can be attributed to the low SFE of the Z-TFMG. These results confirm the non-stick characteristics of the Z-TFMG in terms of oil repellency (i.e., oleophobic properties). F-TFMG was not included in this section of analysis due to its poor cutting characteristics and susceptibility to peeling, as will be shown subsequently.

**Figure 3:**
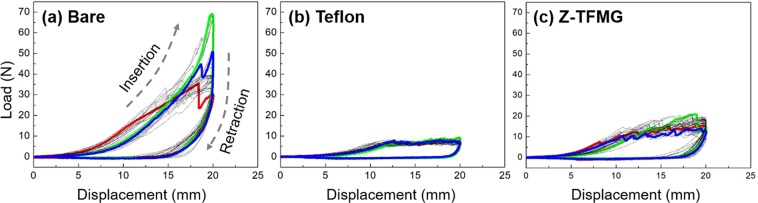
Typical load-displacement curves obtained from (**a**) bare, (**b**) Teflon-, and (**c**) Z-TFMG coated microtome blades upon cutting into pig muscles for 30 times. Red and blue curves respectively represent the first and the last cuttings, while green indicates the cutting having the highest load.

Cutting tests were performed on pig muscle to compare the performance of the Z-TFMG coating with that of the Teflon coating (common used to reduce friction forces) on bare microtome blades. A bare microtome blade was also included in the comparison. Figure 3(a–c) present the load-displacement curves obtained upon the insertion and retraction of the microtome blades (bare, Teflon-coated, and Z-TFMG-coated) into pig muscles a total of 30 times. Insertion force is associated primarily with the effects of cutting and friction, whereas retraction force is associated primarily with the effects of friction^12^. As shown in Fig. 3, the forces generated by the insertion/retraction of Z-TFMG coated blade were lower than those of the bare blade but higher than those of Teflon-coated blade. The forces generated by all samples gradually increased during the first ~10 mm of insertion. With the gradual increase in contact area between the blade and muscle, the friction forces gradually became dominant over the cutting force imposed by the edge of the blade. Following insertion 10 mm into the muscle, the curves from the Teflon-coated blade remained constant at ~9 N, whereas the curves from the bare sample continued a dramatic increase, eventually reaching ~70 N. During the insertion of the Z-TFMG coated sample, the friction forces gradually increased from ~12 N (at 10 mm) to ~24 N (at 20 mm). As shown in Fig. 3(b), the forces generated by the insertion of the Teflon-coated blade plateaued at ~9 N (at 10–20 mm), due to the fact that the resistance from surface friction was very low. This plateau did not appear in the results for the bare microtome. Instead, the cutting force increased significantly with an increase in contact surface due to an increase in surface friction as the microtome moved deeper into the muscle. As indicated by the blue and red curves in Fig. 3(c), forces generated by the insertion of the Z-TFMG coated blade plateaued at roughly ~12 N (at ~10 mm). Overall, the forces imposed by the Z-TFMG coating were slightly higher than those of the Teflon coating; however, they are still very low, particularly when compared to the bare sample. In our previous study involving the insertion of TFMG-coated needles^12^, we observed a similar plateau, indicating that the friction force did not dominate the piercing force as the needle advanced into muscle or rubber.

**Figure 4:**
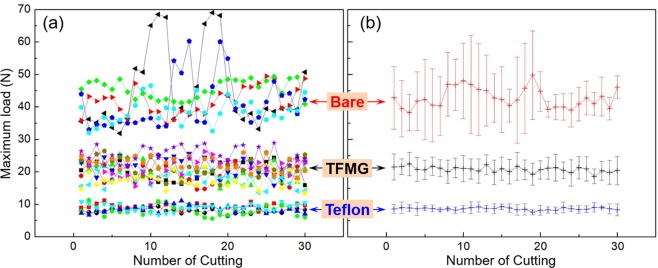
Maximum loads as a function of number of cutting obtained from 5 bare, 5 Teflon-coated, and 10 Z-TFMG coated microtome blades into pig muscles in (**a**) original data and (**b**) average data for each cutting.

**Figure 5:**
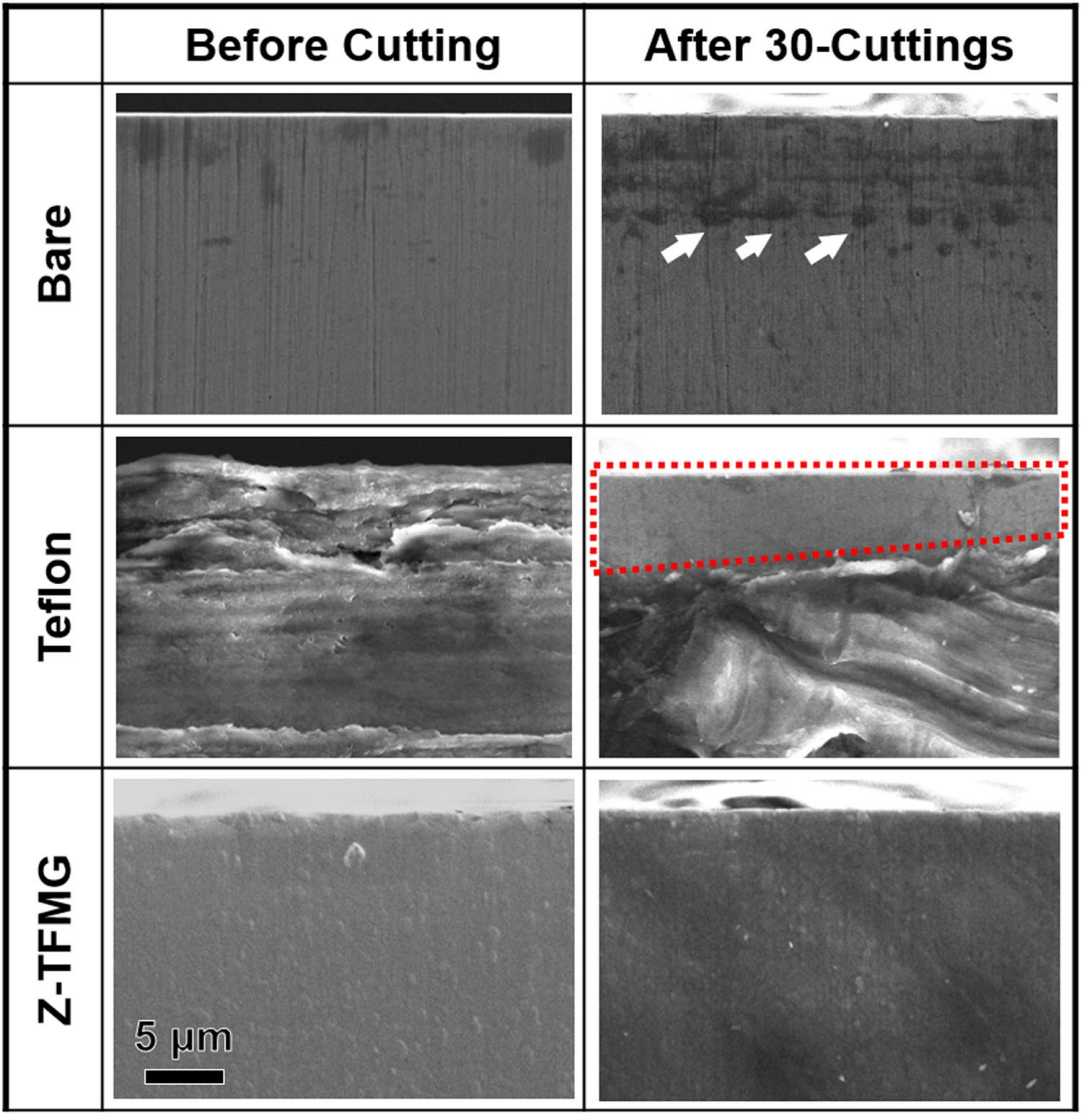
Typical SEM micrographs obtained from edges of bare, Teflon-coated, and Z-TFMG coated microtome blades before and after cutting pig muscles for 30 times. After cutting, white arrows indicate some examples of stains on the bare blade, while red dots outline the coating peel-off region leaving the bare surface exposed in Teflon-coated blade.

Figure 4(a) presents the maximum loads as a function of insertion depth obtained from five bare, five Teflon-coated, and ten Z-TFMG coated microtome blades. The average values obtained from the blades are clearly illustrated in Fig. 4(b). The average highest forces obtained from the samples were as follows: bare blade (42.8 ± 8.2 N), Z-TFMG coated blade (20.8 ± 3.3 N), and Teflon-coated blade (8.63 ± 1.22 N). The cutting force required for the Z-TFMG coated blade was 51% lower than that of the bare blade, whereas the force required for the Teflon-coated was ~80% lower. The standard deviation associated with these averages were as follows: bare blade (19.0%), Z-TFMG coated blade (16.3%), and Teflon-coated blade (14.1%). This variation can presumably be attributed to the inhomogeneous nature of pig muscle and experiment error. These results demonstrate the effectiveness of Teflon coatings in enhancing cutting performance; however, the poor adhesion and toxicity of Teflon limit its applicability as a coating for medical devices. SEM micrographs of these three microtome blades before use and after being used 30 times (Fig. 5) show Teflon peeling off at the edge of blade to expose the bare surface up to several μm from the edge, as indicated by the red dots. Teflon that peels off from cutting instruments is regarded as a potential source of contamination when applied to microtomes used in the preparation of thin-slice samples. A number of stains were observed on the surface of the bare blade after cutting, indicating high permeability. As shown in Fig. 5, there was no difference in appearance of the Z-TFMG coated blade before and after cutting, demonstrating the robust and good adhesion characteristics of the TFMG coating.

The low friction afforded by the Z-TFMG coating allowed the blade to pass through the muscle with less resistance. Pig muscle also contains fluids such as oil and water, which could potentially cause resistance during cutting. As mentioned previously, the Z-TFMG coating allowed the oil to slide across the blade by lowering the friction force (see Fig. 2). These results confirm the non-stick behavior of the TFMG coating and provide key insights into the means by which low SFE and friction forces affect the sharpness of blades. Their subsequent effects on wound healing are detailed in a following section.

### Surface morphology of dermatome blades

Table 1 lists the results of surface roughness measurements of coated and uncoated instruments before and after animal experiments. Before skin harvesting, the surface roughness of dermatome blades coated with Z- and F-TFMG was clearly lower than that of uncoated blades. After skin harvesting, the surface roughness of blades coated with Z- and F-TFMG was still lower than that of uncoated instruments regardless of the cutting distance and whether cutting included hair. The surgical cutting of hairless skin increased the surface roughness of blades coated with Z- and F-TFMG by 8.6% and 14.8%, respectively. When the same procedure was conducted using uncoated blades, we observed a ~70% increase in surface roughness. The surgical cutting of hairy skin increased the surface roughness of blades coated with Z- and F-TFMG by 21% and 23.3%, respectively. The same procedure increased the surface roughness of uncoated blades, by 108%, thereby demonstrating the profound influence that hair can have on surgical instruments.

**Table 1:** Surface roughness measurement results of uncoated/coated dermatome blades before and after skin harvesting.

		Hairless skin, 50 cm		Hairy skin, 10 cm	
		Uncoated	Coated	Uncoated	Coated
Z-TFMG	Before harvesting(nm)	15.4 ± 1.6	13.5 ± 0.6	14.0 ± 1.2	13.0 ± 1.8
	After harvesting (nm)	26.0 ± 1.0	14.7 ± 0.8	29.0 ± 2.7	15.7 ± 2.4
	Percentage of change (%)	69.5	8.6	108.0	21.1
F-TFMG	Before harvesting (nm)	13.2 ± 1.8	12.4 ± 1.0	12.8 ± 1.7	12.6 ± 0.9
	After harvesting (nm)	22.9 ± 2.0	14.2 ± 1.1	26.6 ± 3.0	15.5 ± 1.4
	Percentage of change (%)	74.2	14.9	108.1	23.3

Note: Surface roughness values are an average of measurements from five areas near the edge for a given blade (100 µm × 400 µm each).

**Figure 6:**
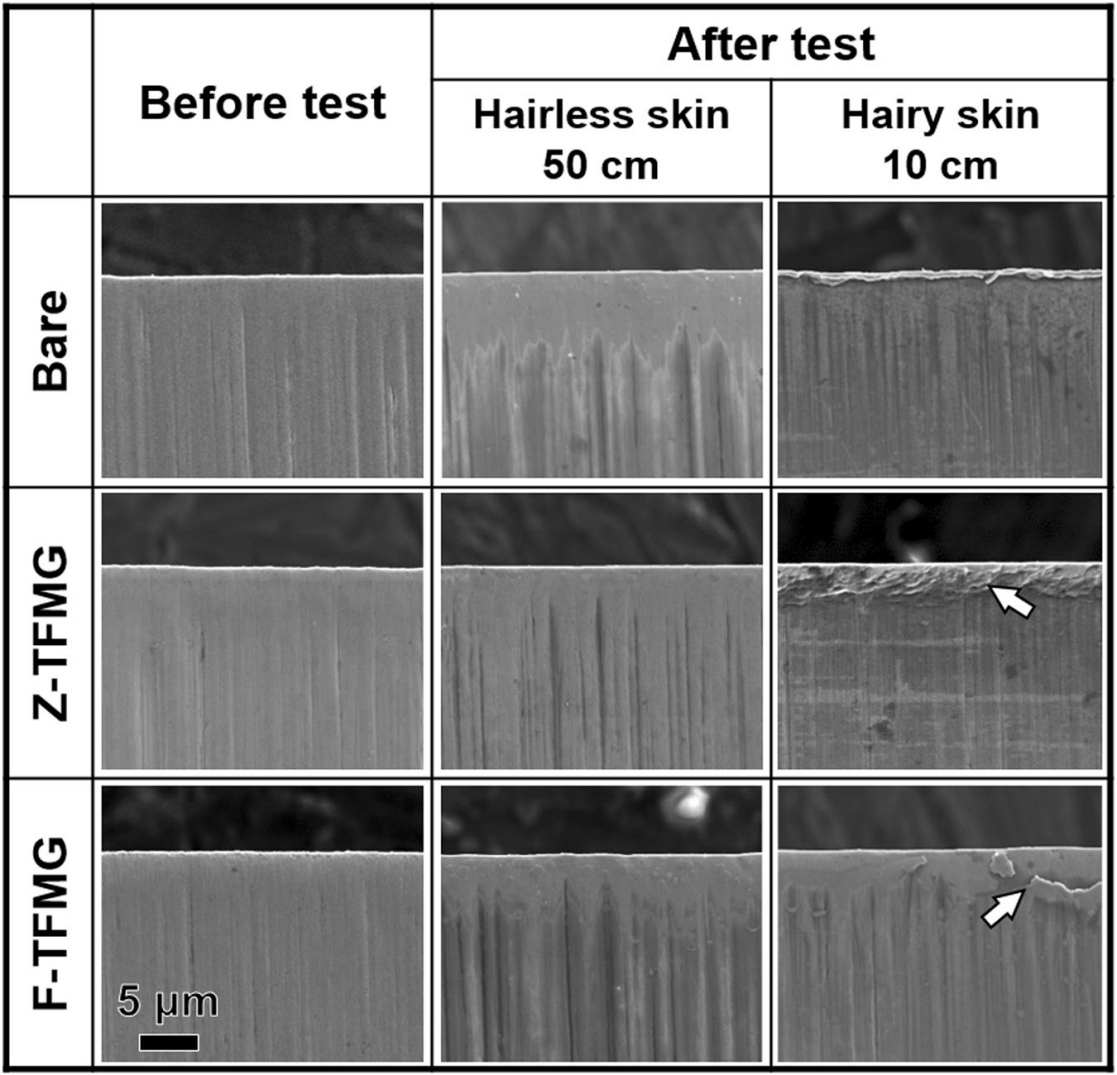
SEM images of bare, Z-, and F-TFMG coated dermatome blades after being used to cut hairless/hairy skin. Z-TFMG coating shows signs of deformation and F-TFMG coating shows signs of delamination after cutting hairy skin (as indicated by white arrows).

Figure 6 presents SEM images of the edge and surface of coated and uncoated dermatome blades before and after skin harvesting. After being applied to hairless skin, the edges of coated as well as uncoated dermatome blades were largely unchanged. Note that the area over which roughness measurements were obtained (100 µm × 400 µm) was far larger than the area covered by the SEM image in Fig. 6. The roughness values presented a notable increase from 15.4 nm before skin harvesting to 26.0 nm after skin harvesting. This indicates that this area suffered from serious wear during the procedure. In contrast, the roughness values obtained from Z- and F-TFMG coated blades did not differ before and after skin harvesting.

After being applied to hairy skin, the uncoated blades showed signs of severe damage, and deformation (i.e., inward curvature) along the edge of the blade. The large increase in roughness values (from 13.2 nm to 22.9 nm) is an indication that hairy skin has a far more dramatic effect on the sharpness of blades than does hairless skin.

This is an indication that uncoated dermatome blades are susceptible to damage under the forces acting on the edge of the blade and the friction force between the sides of the blade and tissue, particularly in the presence of hair. An area of the Z-TFMG surface presented signs of deformation of coating near the edge (as indicated by a white arrow in the figure). The surface of the F-TFMG film showed signs of peeling off (as indicated by the arrow). The Z- and F-TFMG coated blades presented no indication of curvature at the edge of the blades. We believe that the deformation (Z-TFMG) and peeling (F-TFMG) of the films can be attributed to increase resistance when cutting tough hair. Nonetheless, the edges of both coated blades remained far smoother than the uncoated blades after use. The SEM results are in good agreement with the roughness results. Both TFMGs were shown to protect the instruments against damage, even under difficult surgical conditions, as in the presence of hair. The hardness of F-TFMG (12.4 ± 0.1 GPa) exceeded that of Z-TFMG (9.2 ± 0.1 GPa); however, poor adhesion at the edge of the blade caused delamination of the coating under the extreme contact loading associated with cutting. Similar edge delamination was reported in a previous study when F-TFMG coated surgical blades were used to cut soft samples over distances of 25 cm, and widespread delamination occurred when the cutting length reached 50 cm^20^. These previous findings are in good agreement with those obtained in the current work.

### Wound healing after split-thickness skin graft surgery

**Figure 7:**
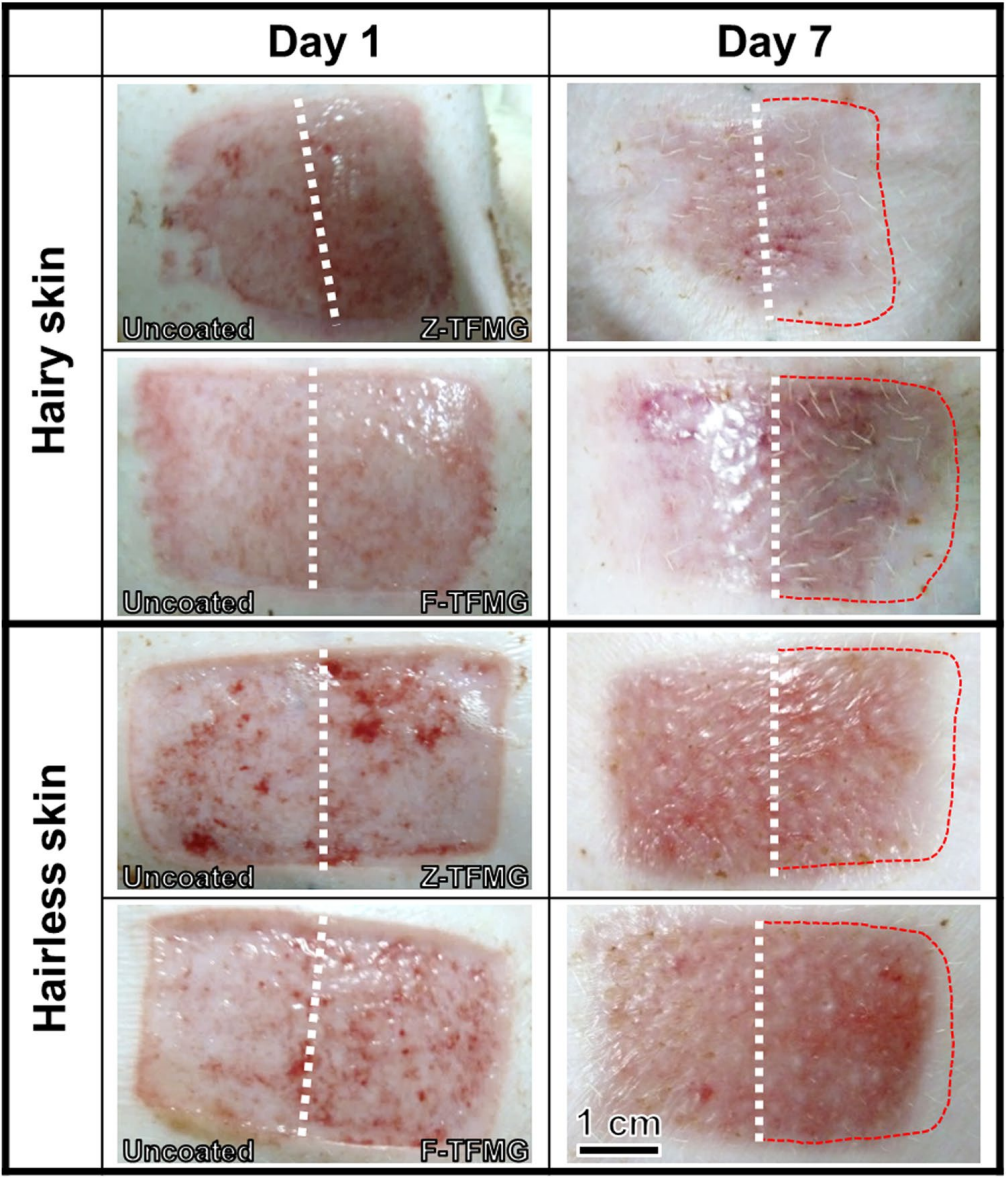
Photographic images of healing wounds between Day 1 and Day 7. The dotted line indicates the division between the areas cut by uncoated blade and the blade coated with TFMG. The red-dotted frames on the right side of the white-dotted line indicate the size of the wound on Day 1 to facilitate a comparison of wound sizes in the various samples.

Figure 7 presents photographic images of healing wounds after various durations of recovery. The wound area to the left side of the dotted line was cut by an uncoated blade, whereas the right side was cut using Z- and F-TFMG coated blades. Normally, the epithelial covering is reconstituted from the edges of the wound and from the cut remnants of hair follicles inside the wounds^9^.

On Day 1, wound healing was the same in the two samples; however, noticeable differences could be observed by Day 7. On Day 7, the area framed by red dots (on the right side) depicts the area of a wound on Day 1. In hairy tissue samples, the wound created by TFMG-coated blades is clearly smaller than that produced by an uncoated blade, indicating that the recovery was more rapid. On Day 7, all of the wounds created in hairless tissue samples were far larger than those in hairy tissue samples, regardless of the cutting instrument employed. This is a clear indication that hairless tissue samples recover more slowly than do their hairy counterparts, due to differences in the physiology of these two types of tissue. Nonetheless, the benefits of using TFMG-coated blades are still evident.

On Day 7, the wounds created using Z-TFMG coated blades in hairless tissue samples were clearly smaller than they were on Day 1. In comparison, the size of wounds created using uncoated blades was nearly unchanged after 7 days. This is a clear indication that Z-TFMG coated blades have a favorable effect on recovery times. On Day 7, the recovery of wounds created in hairless tissue samples using F-TFMG coated blades was less pronounced that in wounds created using Z-TFMG coated blades. Nonetheless, F-TFMG coated blades were still shown to induce faster recovery than were untreated blades. The ability of TFMG-coated blades to withstand the hardness of hair follicles greatly improved cutting effectiveness. We propose here is that the sharper coated blades result in less damage to the wounds, and that this speeds up healing and thereby reduces the likelihood of infection or complications. Nonetheless, further studies will be required to confirm the mechanism underlying the accelerated healing. These benefits are particularly evident when the donor area is on the scalp or when the patient has thick body hair.

**Figure 8:**
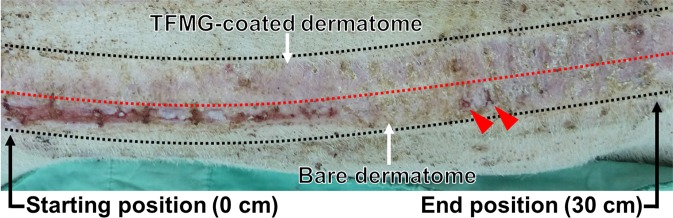
Photographic image of wound healing after 7 days in case of a long distance of 30 cm for hairless skin grafting. The surgery operation using bare blade leads to a severe wound at the initial position of approximately 0~15 cm, and some severe wounds (as indicated by red triangles) are still seen at wound position of approximately 15~30 cm.

**Figure 9:**
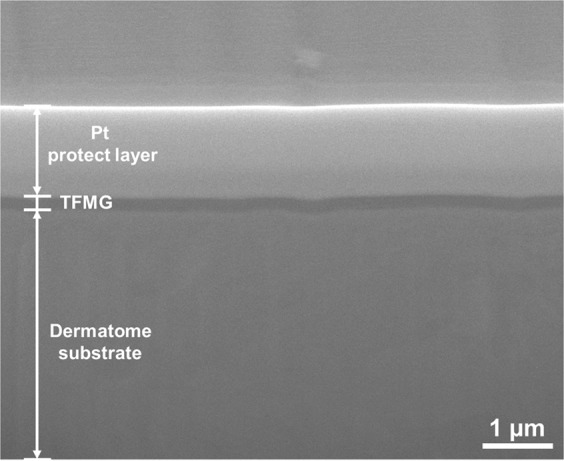
Typical focused ion beam-based cross-sectional SEM image of Z-TFMG-coated blade after it was used to create an incision 30 cm in length.

On the other hand, the wound healing after 7 days in case of a long distance of 30 cm for skin grafting was recorded as shown in Fig. 8. The red dotted line on the photographic image of healing wound separate surgery operation by Z-TFMG (upside) coated and bare (bottom side) blades, respectively. Obviously, skin harvesting using bare blade leads to a severe wound at the initial position of approximately 0~15 cm, and some severe wounds (as indicated by red triangles) still can be recognized at wound position of approximately 15~30 cm. There was evidence of severe wounding in a small section of the incision. Nonetheless, nearly the entire incision produced using a Z-TFMG coated blade recovered more completely within that period of time. This is a clear demonstration that the smooth surface provided by the TFMG coatings reduced the amount of damage, even after being applied over a long distance. Figure 9 presents a cross-sectional SEM image of the Z-TFMG-coated dermatome blade after it was used to create an incision 30 cm in length. This image shows no signs of obvious peeling or abrasion after the long incision.

**Figure 10:**
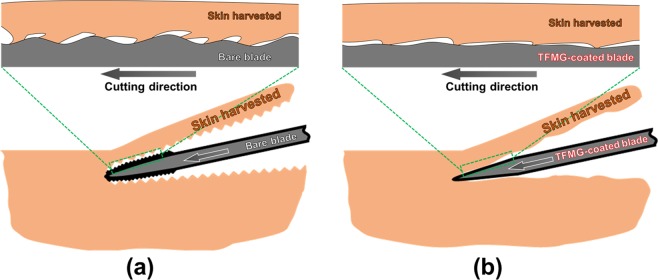
Schematic illustrations of the proposed hypothesis for (**a**) bare and (**b**) TFMG-coated blades during skin harvesting. The rough surface and sticking with tissue are exaggerated for the bare blade to facilitate visual comparison.

We hypothesize that the reduced damage can be attributed to the smoother surface as well as the low COF and SFE of the TFMG-coated surface, leading to enhanced sharpness of the blade edge. McCarthy *et al*.^26^ reported that a smoother surface results in reduced cutting friction so as to enhance sharpness. In addition, low COF and low SFE provide the blade with a non-stick surface, leading to less tearing of the tissue. The proposed hypothesis is schematically illustrated in Fig. 10. In Fig. 10(a), the surface of a bare blade surface tends to adhere to tissue due to high SFE and COF, which ultimately increases the friction forces and causes damage to the tissue. Skin tissue is highly susceptible to tearing by rough and sticky blades. Thus, in most clinical settings, the blade is held within a hand held dermatome device that is electrically operated to provide a steady to-and-fro sawing-like motion in order to harvest the skin graft. The TFMG-coated dermatome blade in Fig. 10(b) has a smooth surface for slicing through tissue to reduce the effects of sticking, and thereby reduce the likelihood of tearing.

Disa *et al*.^27^ demonstrated that the width of scars from incisions created by a sharp scalpel are less pronounced than those created by a rough scalpel. They also showed that incisions created by sharp blades present less inflammatory response during the initial stages of healing. Thus, sharp blades can be used to enhance the quality of grafts while reducing trauma to the skin at donor wound sites, thereby reducing the time required for epithelial healing.

The beneficial effects of TFMG coatings lie in their ability to improve the overall sharpness of the instrument by providing a robust edge and non-sticking surface as well as reducing friction force along the sides of the blade.

## Conclusions

The results of the oil-repellency/sliding tests on kitchen blades in terms of sliding angle and friction forces were as follows: bare blades (31.6°) and (35 µN), Ti-coated blades (20.3°) and (23.7 µN), and Z-TFMG coated blades (16.2°) and (19.2 µN). We also showed the Teflon coating reduced the cutting forces of an uncoated microtome blade by ~80%, whereas the proposed Z-TFMG achieved a ~51% reduction. The Z-TFMG presented no indications of delamination after being used 30 times for cutting; however, the Teflon coating proved highly susceptible to peeling and the bare lade was affected by surface staining. These results demonstrate the efficacy of the TFMG coating in terms of low friction, non-stick performance, and substrate adhesion. The performance of Z-TFMG and F-TFMG was also evaluated in split-thickness skin graft surgery using dermatome blades aimed at elucidating the influence of TFMG coatings on the healing of surgical incisions. When tested on hairless skin, the surface roughness of uncoated blades increased by approximately 70%; however, the surface roughness of Z-TFMG coated blades increase by only 8.6%. The presence of hair in split-thickness skin grafting can severely damage uncoated blades after being used for a distance of only 10 cm. In contrast, Z-TFMG was shown to protect blades during skin grafting surgery by providing a smooth surface morphology to reduce friction force and thereby improving blade sharpness. After 7 days, the wounds created using Z-TFMG coated blades were noticeably smaller than those created using uncoated blades, in both hairless and hairy tissue samples. When making long (30 cm) incisions similar to those used in skin grafting procedures, Z-TFMG coated blades were shown to reduce the recovery time, compared to uncoated blades. These results demonstrate the efficacy of TFMG as a candidate coating aimed at protecting the surface of surgical blades and reducing the friction between the blade and surrounding tissue to facilitate wound recovery.
